# Whole-exome sequencing reveals insights into genetic susceptibility to Congenital Zika Syndrome

**DOI:** 10.1371/journal.pntd.0009507

**Published:** 2021-06-14

**Authors:** Victor Borda, Ronaldo da Silva Francisco Junior, Joseane B. Carvalho, Guilherme L. Morais, Átila Duque Rossi, Paula Pezzuto, Girlene S. Azevedo, Bruno L. Schamber-Reis, Elyzabeth A. Portari, Adriana Melo, Maria Elisabeth L. Moreira, Letícia C. Guida, Daniela P. Cunha, Leonardo Gomes, Zilton F. M. Vasconcelos, Fabio R. Faucz, Amilcar Tanuri, Constantine A. Stratakis, Renato S. Aguiar, Cynthia Chester Cardoso, Ana Tereza Ribeiro de Vasconcelos

**Affiliations:** 1 Laboratório de Bioinformática, Laboratório Nacional de Computação Científica LNCC/MCTIC Petrópolis, Brazil; 2 Laboratório de Virologia Molecular, Instituto de Biologia, Universidade Federal do Rio de Janeiro, Rio de Janeiro, Brazil; 3 Instituto de Pesquisa Professor Amorim Neto, Campina Grande Brazil; 4 Faculdade de Ciências Médicas de Campina Grande, Núcleo de Genética Médica, Centro Universitário UniFacisa, Campina Grande, Brazil; 5 Instituto Fernandes Figueira, Fiocruz, Rio de Janeiro, Brazil; 6 Section on Endocrinology and Genetics, *Eunice Kennedy Shriver* National Institute of Child Health and Human Development, National Institutes of Health, Bethesda, Maryland, United States of America; 7 Departamento de Genética, Ecologia e Evolução Instituto de Ciências Biológicas, Universidade Federal de Minas Gerais, Belo Horizonte, Brazil; INSERM, FRANCE

## Abstract

Congenital Zika Syndrome (CZS) is a critical illness with a wide range of severity caused by Zika virus (ZIKV) infection during pregnancy. Life-threatening neurodevelopmental dysfunctions are among the most common phenotypes observed in affected newborns. Risk factors that contribute to susceptibility and response to ZIKV infection may be related to the virus itself, the environment, and maternal genetic background. Nevertheless, the newborn’s genetic contribution to the critical illness is still not elucidated. Here, we aimed to identify possible genetic variants as well as relevant biological pathways that might be associated with CZS phenotypes. For this purpose, we performed a whole-exome sequencing in 40 children born to women with confirmed exposure to ZIKV during pregnancy. We investigated the occurrence of rare harmful single-nucleotide variants (SNVs) possibly associated with inborn errors in genes ontologically related to CZS phenotypes. Moreover, an exome-wide association analysis was also performed using a case-control design (29 CZS cases and 11 controls), for both common and rare variants. Five out of the 29 CZS patients harbored known pathogenic variants likely to contribute to mild to severe manifestations observed. Approximately, 30% of affected individuals carried at least one pathogenic or likely pathogenic SNV in genes candidates to play a role in CZS. Our common variant association analysis detected a suggestive protective effect of the rs2076469 in *DISP3* gene (p-value: 1.39 x 10^−5^). The *IL12RB2* gene (p-value: 2.18x10^-11^) also showed an unusual distribution of nonsynonymous rare SNVs in control samples. Finally, genes harboring harmful variants are involved in processes related to CZS phenotypes such as neurological development and immunity. Therefore, both rare and common variations may be likely to contribute as the underlying genetic cause of CZS susceptibility. The variations and pathways identified in this study may also have implications for the development of therapeutic strategies in the future.

## Author summary

Since the beginning of Zika virus outbreak in Brazil, five years ago, we still don’t understand the genetic factors associated with the small number of babies born with Congenital Zika Syndrome (CZS). Here, we focused on the host genetic susceptibility by studying the whole-exome of the CZS affected (n = 29) and healthy (n = 11) neonates, both born to ZIKV infected women from Brazil. We applied two strategies: 1) Determine whether cases individuals have pathogenic or harmful variants that explain the CZS outcomes (i.e. microcephaly) independently of ZIKV infection or not, 2) Exploring the common and rare variants association with CZS. We found that common and rare variants in genes like *DISP3* and *IL12RB2* could explain some level of the susceptibility to CZS. Moreover, by considering these and other candidate genes, we observed an over-representation of Gene Ontology terms related to neurological system, metabolism and microtubule-cytoskeleton organization.

## Introduction

Novel and recurrent viral outbreaks are of enormous concern for global health management as it can cause life-threatening illness and severe economic damage. Latin American countries are at increased risk of spreading viruses due to favorable environmental conditions and limited resources to fight infections. In the past few years, Brazil has been struggling with several arboviral outbreaks such as Yellow fever, Dengue, Zika, and Chikungunya [1]. Specifically, Zika virus (ZIKV) infection has been under the spotlight due to the association of its vertical transmission with microcephaly and even death of fetuses during pregnancy [2–6]. These outcomes and other neurological impairments are part of the so-called Congenital Zika Syndrome (CZS) [7].

CZS is a complex phenotype and several risk factors may contribute to its susceptibility and severity. These include factors related to the virus itself, to the environment, and to maternal and children’s genetic background. Viral-related factors include ZIKV genetic diversity and coinfection events with chikungunya virus (CHIKV) [8] or previous dengue virus (DENV) [9]. Among maternal factors, it was described that gestational age at the moment of ZIKV infection, the immune response at the maternal-fetal interface [10] and genetic variation in adenylate cyclases [11] and *TLR3* [12] genes are associated with CZS outcomes.

Transcriptome- and exome-wide analyses of dizygotic twin pairs, discordant for CZS, revealed differential gene expression signature in mTOR and Wnt pathways, both involved in cell proliferation and cell migration processes [13]. Polymorphisms of collagen-family genes and extracellular matrix alterations were also found in postmortem brains of CZS neonates, suggesting an underlying molecular mechanism for neurological malformations [14]. A preliminary analysis has also suggested a role for TNF polymorphisms in severe microcephaly [12]. However, most of the studies cited above include a small sample size and a clear role for genetic variations in children and CZS still remains to be determined.

**Fig 1:**
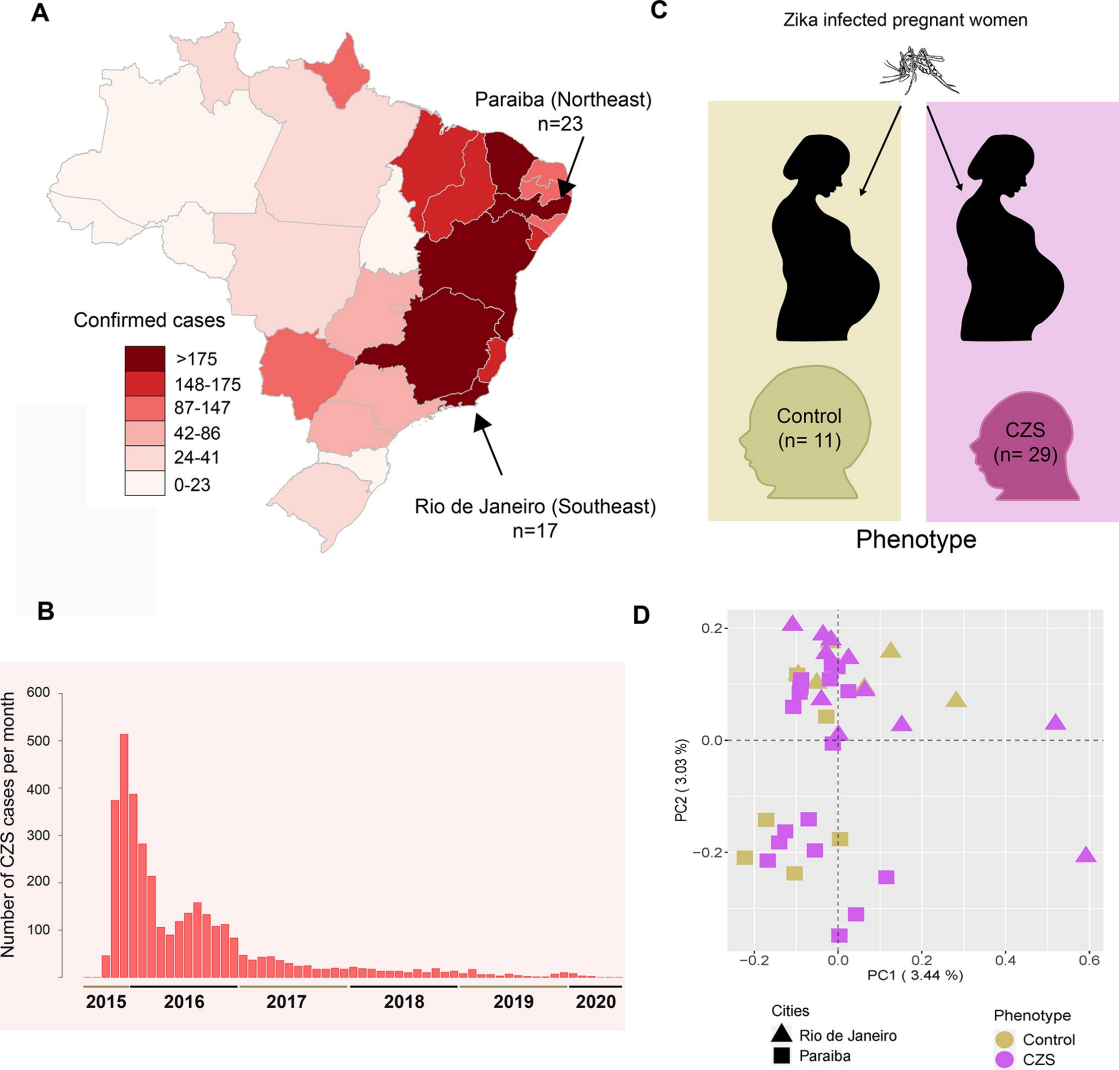
Geographical and temporal distribution, and Population Structure Analysis of Congenital Zika Syndrome (CZS) cohorts from Northeast and Southeast Brazil. (A) The map shows the accumulated distribution of CZS cases and the states in which the individuals were sampled (Coordenação-Geral de Vigilância das Arboviroses, 2020). In the Northeast, individuals were sampled in Paraiba state and in Southeast, individuals were sampled in Rio de Janeiro. (B) Bar plots show accumulated temporal distribution of CZS cases from the first outbreak in Brazil (2015) until present. (C) The cartoon shows the sampling design for cases and controls. Both types of individuals were sampled from ZIKV infected mothers. (D) Principal Component Analysis of the exome data of 40 Brazilian individuals with and without Congenital Zika Syndrome. Purple and yellow forms represent CZS and control individuals, respectively. Squares and circles represent Northeast (Paraiba) and Southeast (Rio de Janeiro) individuals, respectively.

From 2015 to 2020, 3,534 cases of CZS were confirmed in Brazil, including 239 stillborn [15]. This number may be even higher considering the suspected cases. According to the Brazilian Ministry of Health data, most of the cases were found in Northeast (56%) and Southeast (26.7%) regions of the country (Fig 1A). Nearly half of cases were observed during late 2015 and 2016, decreasing after late 2017 (Fig 1B). In this work, we explore the genetic basis of susceptibility to CZS in newborns by analyzing the whole-exome data of 40 unrelated infants, originated from the most affected regions of Brazil, exposed to ZIKV during pregnancy and presenting different clinical neurological outcomes (Fig 1C). We analyzed common and rare variants aiming to determine pathogenic or likely pathogenic variants that could explain the CZS phenotype. Furthermore, we seek genetic association with CZS in children exposed to ZIKV during pregnancy. To our knowledge, this is the largest cohort of children exposed to ZIKV infection sequenced to date.

## Methods

### Ethics statement

This study was approved by the Institutional Ethical Committee number 52888616.4.0000.5693 and 52675616.0.000.5269. All patients agreed to participate and signed a consent form.

### Sampling and phenotypic information

During the first outbreak of ZIKV in Brazil (July 2015—June 2016), pregnant women with acute febrile illness with a rash, fetal CNS abnormalities at prenatal ultrasonography, or postnatal microcephaly or other CNS malformation that was believed to be characteristic of congenital infection were referred to the Microcephaly Reference Center Instituto de Pesquisa Professor Amorim Neto (IPESQ) in Campina Grande (Paraíba, Brazil) or Instituto Fernandes Figueira–Fiocruz (Rio de Janeiro, Brazil). Detailed demographic, medical, and prenatal history information, as well as clinical findings, was entered into case report forms by multidisciplinary medical teams. The onset symptoms included fever, exanthema, arthralgia, conjunctivitis, and headache in the pregnant women during gestation. All women were referred for at least one fetal ultrasonography during gestation and magnetic resonance imaging. Just after birth, the cephalic perimeter was measured and the percentile was calculated according to the expected for the gestational age 1. Microcephaly was confirmed at the birth by measuring the cephalic perimeter in which the head circumference was less than 2 SDs for gestational age in most infants. Postnatal head computed tomography was also performed.

ZIKV infection during pregnancy was confirmed in the mothers or babies by serological tests (ELISA IgM/IgG) and RT-qPCR analysis targeting the *env* virus gene [16] (S1 Table). ZIKV RNA was detected in fluid samples, including blood, urine, amniotic fluid obtained by amniocentesis during gestation, or in other fluids after birth (amniotic fluid, cord blood, or both) [14].

Dengue and chikungunya, arboviruses that circulate in the same area, and other congenital pathogens (syphilis, cytomegalovirus, herpes virus 1/2, Toxoplasma gondii, and rubella) were excluded in all cases by IgM / IgG serological enzyme-linked immunosorbent assay (ELISA).

After birth, 29 children that showed microcephaly or other neurological abnormalities (brain calcifications, ventriculomegaly, cortical malformations, agyria/ lissencephaly, congenital contractures and ocular abnormalities) were considered as cases and 11 children without any CZS outcome were considered as controls (Tables 1 and S1). All participants were exposed to ZIKV infection during pregnancy confirmed by RT-qPCR or/and serology.

**Table 1:** Clinical and demographic characteristics of the study cohort.

	CZS cases (N = 29)	Controls (N = 11)
**Sex**		
Male	11 (38%)	3 (27%)
Female	18 (62%)	8 (73%)
**Trimester of exposure to ZIKV**		
First trimester	19 (65.5%)	3 (27%)
Second/third trimester	7 (24.2%)	7 (64%)
Unknown	3 (10.3%)	1 (9%)
Death	8 (27,6%)	0

CZS = Congenital Zika Syndrome.

### DNA extraction and whole-exome sequencing analysis

DNA samples were obtained from peripheral blood using QIAmp DNA Mini Kit (QIAGEN), according to the manufacturer’s instructions. Library preparations were performed using three different kits (S1 Table): Illumina TruSeq Exome, Agilent and Roche sequencing kits according to the manufacturer’s protocols. For TrueSeq and Agilent libraries, we performed the sequencing using Illumina NextSeq 500/550 High Output Kit v2 (150 cycles), generating 2x75 bp paired-end reads. For Roche library, we used Illumina Hiseq X Ten.

Raw data files were processed separately for each sample. Short reads were mapped to the human reference genome (GRCh38/hg38) using Bowtie2 version 2.3.4.1 [17]. The output files in SAM format were converted to BAM files, sorted and filtered by MAPping Quality (MAPQ > 30) using samtools version 1.3 [17,18]. Duplicated reads were later identified using MarkDuplicates from Picard software version 2.18 (http://picard.sourceforge.net/). Single Nucleotide Variants (SNVs) and small insertion and deletions (INDELS) calling was performed using the HaplotypeCaller tool from Genome Analysis Toolkit (GATK) version 4.1 [19] with a combined dataset. Best practices steps for variant calling were followed, including variant quality filtration and base recalibration according to the GATK protocols [20]. SnpEff and SnpSift software version 4.3 [21] and the Ensembl Variant Effect Predictor (VEP) [22] were used to predict genetic effects and molecular impacts of the variants called. Next, dbGWAS [23] and ClinVar [24] information were used to identify variants previously associated with microcephaly phenotype as well as those with clinical significance. The Minor Allele Frequency (MAF) was annotated for each variant according to the global variant frequencies in dbSNP [25], 1000Genomes [26], ExAC [27], GnomAD [27].

After merging all exome sequences, missing data in at least two individuals and variants out of the Hardy-Weinberg equilibrium (1 x 10^−6^ threshold for cases and 1 x 10^−10^ for controls) were excluded from our analysis using PLINK [28]. We test for relatedness by inferring the kinship coefficient with REAP [29] (S1 Text). Varsome (https://varsome.com/) was used to annotate pathogenicity status according to ACMG classification and interpretation of clinical genetic variant effects. Raw data of this study is publicly available in SRA-NCBI (www.ncbi.nlm.nih.gov/sra), SRA accession PRJNA655497 and PRJNA517145.

### Potentially pathogenic variants in genes associated with CZS phenotypes

In order to identify variants with a potential pathogenic effect in our cohort, we interrogated variants with deleterious profiles according to computational predictors (SIFT, PolyPhen, CADD and LoFtool). Potentially pathogenic variants refer to SNVs found using computational predictors criteria that have not yet been clinically validated. We selected variants with zigozity profiles in each patient consistent with the phenotypic inheritance pattern of genes related to symptoms characteristic of CZS. We identify these genes by querying the Human Phenotype Ontology (HPO; https://hpo.jax.org/) database (S1 Text) [14]. Then, we prioritize rare nonsynonymous variants (MAF < 5%) and sort out those with a deleterious profile according to computational predictors.

### Association analyses

#### Population structure and confounding variables

To infer population structure and ancestry proportions, we performed a Principal component analysis (PCA) using SNPRelate [30] and a genetic clustering using ADMIXTURE [31] (S1 Text). To determine if population structure or a non-genetic variable explain the differentiation between cases and controls, we performed association analyses among the phenotype (CZS/Control) and 10 first principal components (Fig 1D), ancestry proportions, sex, and timing of gestational exposure to ZIKV (S1 Text). CZS cases and controls were compared using Fisher exact tests for categorical variables (Sex and the timing of gestational exposure to ZIKV) and Wilcoxon rank-sum tests for continuous variables (ancestry proportions and principal components).

#### Variant-based association analyses

For this approach, we performed a MAF filter (MAF > 5%) on our dataset. Using this dataset, we tested for association between each common variant with CZS using a Firth’s logistic regression. We applied the Firth’s logistic regression due to our small and unbalanced sample size [32], avoiding the traditional logistic regression because of the need for more than 500 individuals to have a good inference of parameters [33]. Moreover, considering our sample size and a minor allele frequency of 0.3, the minimum OR value to achieve power of 80% under an additive model would be 7.5 for risk effect and 0.13, considering a protective effect.

The exome-wide analysis was performed under an additive model using the R package logistf. Variants with p-value < 5 x 10^−4^ were also analyzed under a dominant model. R scripts for running and plotting the logistic analyses are freely available on https://github.com/vicbp1/Genetic-Arquitecture-of-Zika.git.

#### Gene-based association analyses

Due to the potential to cause disease, we also focused on rare nonsynonymous variants in coding regions. We investigated whether the patterns of accumulation and distribution of genes harboring more rare variants have an effect on the CZS phenotype. Genes with less than three rare variants were filtered out according to Dutta et al. [34]. We applied the C-alpha and SKAT approaches and set a stringent significance threshold at 8 × 10^−6^ corresponding to the Bonferroni adjustment for 6,219 genes (S1 Text). SKAT approach was run with and without adjustment for covariates (S1 Text). Finally, in order to associate the biological relevance of mutated genes to the patient’s clinical outcome, we performed an enrichment analysis using Gene Ontology and KEGG in clusterProfiler [35], and Reactome in ReactomePA package [36].

## Results

### Whole-exome sequencing analysis

The whole-exome sequencing (WES) performed in the 40 patients reached, on average, 98% of the reads mapped to the reference genome. Targeted exonic regions achieved a mean of coverage of 125-fold with a per-base depth greater than 20-fold in 92% of the sites (S1 Table). We were able to identify a total of 378,649 SNVs across the 40 individuals. Among the variants targeting coding regions, 46% of them were missense, followed by synonymous (42%), splice sites (9%), frameshift (1%), insertions and deletions in frame (1%), and nonsense (1%) variations. Next, we separated the variant table into two distinct dataset containing common SNVs in our cohort and rare variations according to the population databases, respectively. The S1 Fig describes the workflow followed in the present study. After removing low-quality SNVs and filtering out missing data, we selected 144,153 sites with allele frequency greater than 5% in our cohort to be used in the association analysis based on a case-control design (29 CZS cases and 11 controls). The second dataset included 38,361 rare nonsynonymous SNVs with MAF < 5% in GnomAD and 1000Genomes (S2 Fig) in order to perform a gene-based association analysis and prioritize pathogenic variants.

### WES analysis unveiled known germline pathogenic variants across CZS patients

Three well-established germline pathogenic variants were identified in three genes known to cause genetic disorders. All variants were found in heterozygosity with the wild type allele in genes previously associated with phenotypes supporting a dominant effect. The amount of read sequences carrying the pathogenic allele in each site was greater than 90 reads for all patients. Two CZS-affected individuals, CZS_8 and CZS_27, both females, carried the variant rs1050828 (MAF _gnomAD_: 0.009) in *G6PD* gene associated with hemolytic anemia due to G6PD deficiency (OMIM #300908) in an X-linked dominant manner. Furthermore, two affected males, CZS_18 and CZS_25, carried a pathogenic variant (rs61755320; MAF _gnomAD_: 0.0029) that promotes an Alanine to Valine modification in the *SPG7* gene causing spastic paraplegia 7 (OMIM #607259). In the affected individual CZS_28, a female patient, we reported the missense variant rs118192168 (MAF _gnomAD_: 0.00001) in *RYR1* gene previously associated with susceptibility to malignant hyperthermia (OMIM #145600), central core disease (OMIM #117000), and minicore myopathy with external ophthalmoplegia (OMIM #255320). Therefore, known pathogenic mutations (i.e. those previously reported as pathogenic in the databases used) are likely to contribute to mild to severe manifestations observed in five out of the 29 cases.

### Potentially pathogenic variants in genes associated with CZS phenotypes

**Fig 2:**
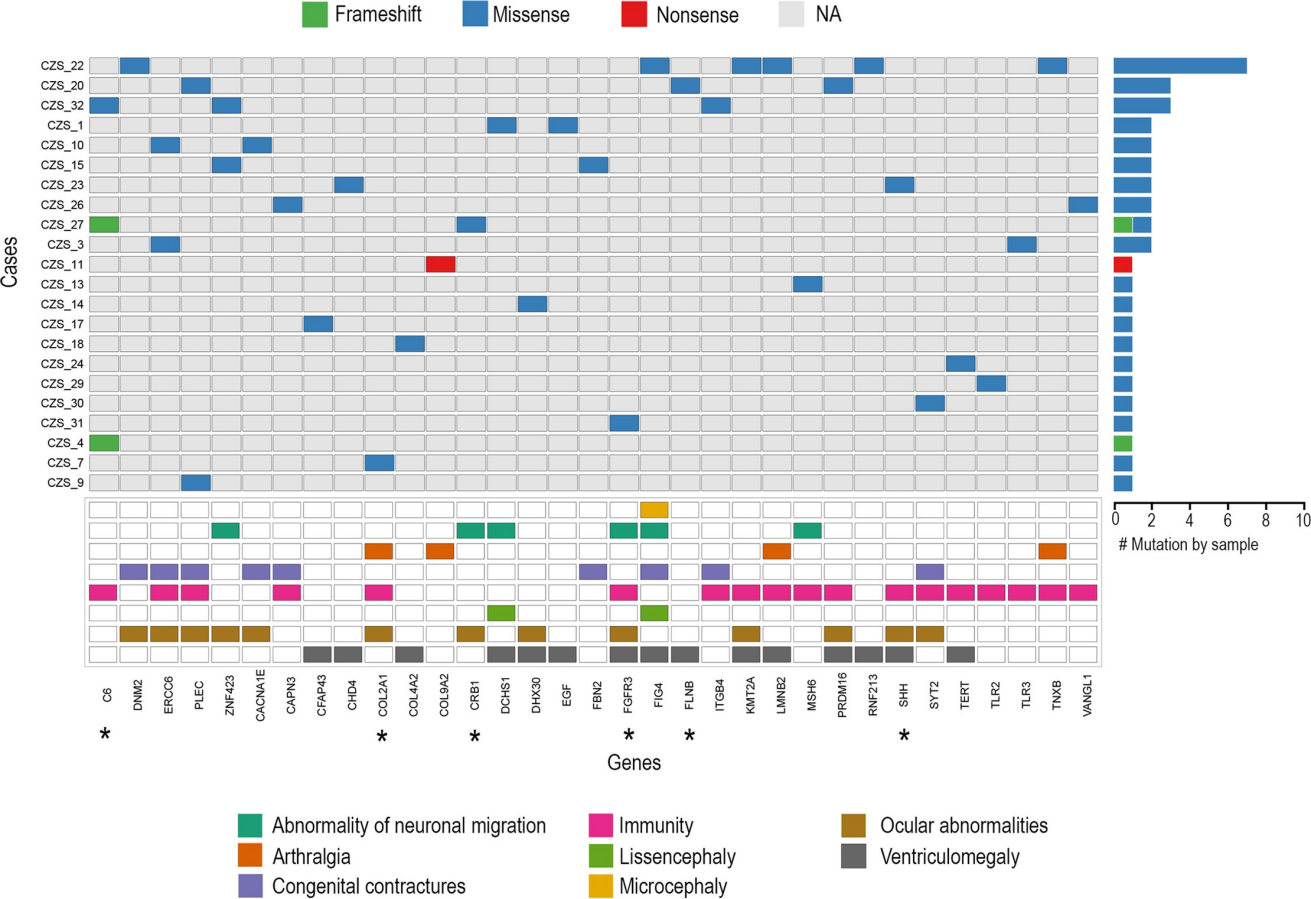
Co-occurrence of rare non-synonymous variants in genes associated with CZS phenotypes. Comut plot representation of harmful genetic variants in a series of 22 newborns with CZS. (Right) Frequency of variants per patient ranked by the number of mutations. (Middle) Heat-map of variants in each patient for an individual gene by the type of mutation. (Bottom) Human Phenotype Ontology term associated with each gene. Asterisk indicates genes with likely pathogenicity variants.

Our search for novel candidate pathogenic variants was focused on a list of genes ontologically related to common phenotypes observed in CZS (S1 Text). We identified 38 rare SNVs computationally predicted as potential candidates to cause damage spread across 33 genes (S2 Table). All variants were heterozygous in genes associated with autosomal dominant phenotypes, being most of them classified as uncertain significance following the ACMG/AMP standards rules for classification of genetic variants [37]. The *C6* gene showed the greatest number of likely pathogenic variants among the highlighted genes. Patients CZS_4 and CZS_27 shared the same rare SNV (rs375762365, MAF _gnomAD_:0.00188) characterized as likely pathogenic in *C6* gene. We also observed a large number of deleterious variants in the affected individual CZS_22 (Fig 2 and S2 Table).

We found two pathogenic variants in the *CAPN3* (rs80338802; MAF _gnomAD_: 0.00003) and *FIG4* (rs121908287; MAF _gnomAD_ 0.00098) genes in patients CZS_26 and CZS_22, respectively. The heterozygous missense variant found in *CAPN3* was previously associated with muscular dystrophy, limb-girdle, autosomal recessive 1 (OMIM #253600). However, autosomal dominant forms of the disease were also associated with variants in this gene. The rs121908287 in the *FIG4* gene was reported as related to Charcot-Marie-Tooth disease, type 4J (OMIM #609390) when found in compound heterozygosity with a second *FIG4* pathogenic variant. Nevertheless, we did not observe the second pathogenic allele in the CZS_22 patient.

Seven likely pathogenic variants were identified in six genes (*COL2A1*, *CRB1*, *FGFR3*, *FLNB*, *SHH* and *C6*), being six of them missense and one frameshift (Fig 2 and S2 Table). We also found 12 novel variants in *FGFR3*, *FBN2*, *TNXB*, *KMT2A*, *CHD4*, *COL4A2*, *SYT2*, *ZNF423*, *RNF213*, *DNM2*, and *LMNB2* genes of seven CZS patients (S2 Table). With exception made for patients CZS_25 and CZS_27, that carried both known and likely pathogenic mutations previously described, more than 30% (n = 10/29) of the affected individuals harbored at least one harmful SNV classified as pathogenic or likely pathogenic in genes related to CZS.

### *DISP3* and *IL12RB2* as likely candidate genes associated with CZS

Previous sections showed that some pathogenic variants could mislead our association analysis. For this reason, association tests were conducted using the complete dataset of patients (n = 40) and a subset (n = 35) by excluding five CZS individuals with known pathogenic SNVs. We performed association analysis at two levels: variant-based and gene-based analyses. Due to our small sample size, we did not reach the statistical significance for a typical genome-wide analysis (5 x 10^−8^). However, by analyzing the differentiation between affected and healthy individuals, we identified candidate associations among the CZS outcome and SNVs and genes.

After testing for association between the phenotype with ancestry proportions and non-genetic variables, we observed a significant association between the timing of gestational exposure to ZIKV (p-value: 0.03, S3 Fig and S3 Table), which was included for adjustment in the common and rare variant analyses. Moreover, we detected some level of population structure on the sample related to the geographical origin but apparently not to the CZS condition (Figs 1D and S3).

**Fig 3:**
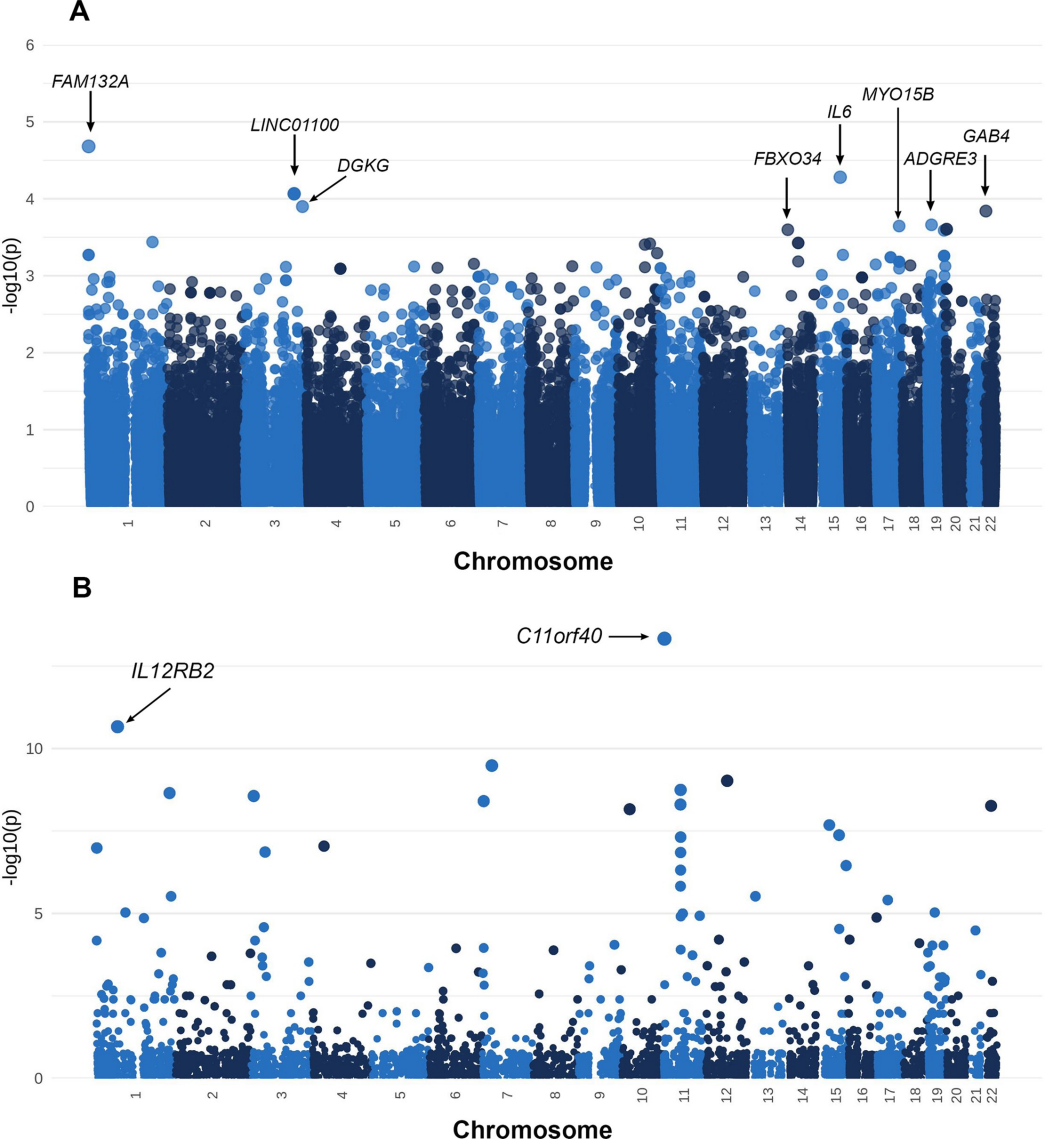
Association analyses of common and rare variants. (A) Manhattan plot of the Firth’s logistic modelling for common variants of the CZS dataset (n = 35). We modeled the parameters considering the binary response (CZS / Control) and the genetic variants while adjusting for the gestational exposure to ZIKV in the mother. Each point corresponds to a genetic variant. (B) Manhattan plot of the results of the C-alpha test of the CZS dataset (n = 35), we determine the differential distribution and accumulation of rare variants grouped in genes between cases and controls. Each point represents a gene.

After removing SNVs with MAF below 5% in our cohort, we kept 144,153 variants. Results of the Firth’s logistic regression under the additive model are shown in Fig 3 (See also S4 and S5 Figs and S4 and S5 Tables). None of the variants reached a genome-wide significance level (lowest adjusted p-value: 1.16 x 10^−5^). Moreover, the inflation in analysis due to population structure was not observed (Figs 3, S4 and S5, λGC = 0.903). Interestingly, both *FBXO34* (rs1045002; MAF _gnomAD_: 0.3837; Odd ratio: 0.041; 95% CI: 0.0003–0.3081) and *MYO15B* (rs820152; MAF _gnomAD_: 0.3219; Odd ratio: 0.1294; 95% CI: 0.0229–0.4385) showed missense mutations with protective effect (S4 and S5 Tables). Overall, 27 variants of 17 genes were associated with CZS under a suggestive threshold of 5 x 10^−4^ (S4 Table). When these variants were analyzed under a dominant model, a strong protective effect was observed for rs2076469 with higher significance, at *DISP3* gene (S4 and S5 Tables, MAF _gnomAD_: 0.2375; Odd ratio: 0.0126; 95% CI: 0.0001–0.1223; lowest adjusted p-value < 6.32 x 10^−6^).

Next, we investigated whether the patterns of accumulation and distribution of rare variants in SNP sets were different among cases and controls. We applied the C-alpha test [38] and SKAT Binary [39] to analyze 33,417 rare nonsynonymous (MAF < 5%) variants across 6,219 genes. We found that the *IL12RB2* gene showed the most significant unusual distribution of rare variants in our cohort for both approaches (lowest p-value _C-alpha_ < 2.18x10^-11^ and lowest p-value _SKAT Binary Adjusted_: 4.1x10^-4^; S6, S7 and S8 Tables and Fig 3B). Interestingly, most of the rare alleles in this gene were observed in controls. Both results from common and rare analyses were in congruence with the CZS reduced dataset results.

### Enrichment analysis unveils variations in biological pathways associated with CZS

We investigated the biological significance of the genes prioritized in our analyses, including those with harmful SNVs and harboring common and rare variants highlighted by association analyses. We performed functional enrichment analysis using GO terms, KEGG, and Reactome. Gene ontology over-representation approach revealed 20 GO terms significantly enriched in our gene set (p-value < 0.05; Bonferroni method; S9 Table) related to extracellular matrix organization, transmembrane receptor protein serine/threonine kinase signaling pathway, calcium ion transport, nervous system development and regulation of Wnt signaling pathway (S9 Table). Alterations in collagen family genes significantly contributed to enrichment in pathways linked to assembly and degradation of extracellular matrix organization through collagen fibrils formation (S10 Table). We also found differentiation in immune system pathways associated with the TLR signaling cascade due to mutations in *TLR2* and *TLR3* genes. Furthermore, nine mutated genes (*CHD4*, *COL2A1*, *COL4A2*, *COL9A2*, *EGF*, *ITGB4*, *TERT*, *TLR3*, *TNXB*) belonged the same pathway of the Human papillomavirus infection (hsa05165) according to KEGG over-representation analysis. Focal adhesion, ECM-receptor interaction, and PI3K-Akt signaling pathway were also significantly enriched in our analysis (S11 Table).

## Discussion

The risk of CZS has been a major concern for women exposed to ZIKV infection early in pregnancy [40,41]. Previous studies showed that genetic and epigenetic factors, such as DNA variations and maternal protein malnutrition, may contribute to this disorder [10,11,42]. Thus, to address genetic variation and susceptibility to life-threatening neurodevelopmental dysfunctions in CZS patients, we carried out an exome-wide screening in 40 children with and without CZS born to ZIKV infected mothers. To date, this cohort comprehends the largest group of ZIKV infected patients ever sequenced using a high-throughput approach. We identified candidate variants in genes that could play a protective/deleterious role for CZS susceptibility.

Many monogenic disorders may overlap common symptoms found in CZS. Thus, identifying pathogenic variants at individual level are crucial to avoid misclassification of children with mendelian phenotypes as CZS cases. Indeed, well-established germline pathogenic mutations were identified in five cases. These mutations were likely to contribute to the clinical symptoms observed, since the inheritance pattern of the corresponding genetic disorder was dominant and the patients were heterozygous. Mutations in *SPG7* and *RYR1* genes are associated with muscle developmental impairment causing paraplegia and myopathy, respectively [43]. Both phenotypes challenge the CZS diagnosis in these patients due to possible overlapping effects. Also, deficiency in *G6PD* gene seems to enhance susceptibility to viral infection [44–46]. Therefore, further association analyses were also conducted excluding these individuals to avoid a possible confounding, and similar results were found.

Host inter-individual variations in genes responsible for human leukocyte antigen (HLA), innate immunity and cell receptors greatly contribute to differences in the clinical course of viral infection among patients [47,48]. Genes included in these categories usually undergo differential expression profiles during ZIKV infection mostly associated with extracellular matrix, cell adhesion, collagen-encoding as well as mTOR and Wnt pathways [13,14,49]. Interestingly, two genes reported in our analysis, *FGFR3* and *ITGB4* (S2 Table), were upregulated in the brain of patients with CZS [14]. Both are important in the regulation of bone development and cell-cell adhesion. We found a novel harmful variant in *FGFR3* classified as likely pathogenic according to ACMG criteria. Heterozygous mutations in this gene are known cause of achondroplasia (OMIM #100800), hypochondroplasia (OMIM #146000) and others congenital abnormalities. Likely pathogenic variants were also identified in *COL2A1*, *CRB1*, *FLNB*, *SHH*, and *C6*. Overall, these genes were mostly linked to disorders of immunity, ocular abnormalities, ventriculomegaly and abnormality of neuronal migration. Our analysis enabled the identification of rare variations that might help to explain the clinical phenotypes, but with no impact on the association analysis due to their low frequencies.

We were unable to find associations among common variants at a genome-wide significance level due to the small sample size. However, results of Firth’s logistic regression analyses under an additive model showed a protective effect for variants at *FBXO34*, *TACC2*, *MYO15B* and *DISP3* genes when a suggestive significance level (5 x 10^−4^) was adopted. This threshold, although arbitrary, helps us to select the highly differentiated variants between cases and controls. Despite these variants not reaching the statistical significance for a typical genome-wide analysis (usually 5 x 10^−8^), they showed clear differences in the allelic frequencies between cases and controls and are probably valuable candidates for further investigation. Additionally, under a dominant model, we detected a suggestive association for SNV rs2076469, at *DISP3* gene, which is highly expressed in neural tissue with impact on the proliferation and differentiation of neural precursors [50].

Furthermore, our gene-based analyses showed the *IL12RB2* gene as the most significant results after adjustment for covariates and multiple comparisons. This gene encodes a transmembrane protein, corresponding to a subunit of interleukin 12 (IL-12) receptor. IL-12 regulates natural killer (NK) responses, differentiation of Th1 cells and induces interferon-gamma production. NK cells are important in early response against intracellular pathogens like ZIKV or CHIKV [51], being detected in the fetal liver as early as at 6^th^ week of gestation [52]. Recently, Messias *et al*. [49] demonstrated that IL12β subunit was upregulated in ZIKV infected thymic epithelial cells. These results suggest a possible role for IL-12 signaling in early-response to ZIKV infection.

Our findings suggest an over-representation of pathways involved in proliferation of neural stem cells and viral-induced infection such as PI3K-Akt signaling pathway. In addition, accumulation of damage in biological pathways required for regulation of cellular homeostasis (e.g focal adhesion and protein digestion and absorption) through variations in structural and functional proteins such from collagen family seems to be the underlying mechanism of ZIKV infection. Collagen genes (COL) are part of the extracellular matrix and are essential for the development of the brain and the blood-brain barrier [53]. It was suggested that *COL* genes interact with some virus during infection [54]. Moreover, Aguiar *et al*. [14] demonstrated the upregulation pattern of COL genes in microcephalic children associated with ZIKV infection during pregnancy [14]. We also found differentiation in immune system pathways related to the TLR signaling cascade due to mutations in *TLR2* and *TLR3* genes. Santos *et al*. (2019) firstly described the association between the rs3775291 in *TLR3* and CZS cases [12].

The main limitation of our study was the small sample size, which reduces our power to detect associations at a genome-wide significance level and also reduces precision of the effect estimates. Therefore, replication studies in independent cohorts are still required to validate the associations observed. Nonetheless, this is the largest cohort of children with the rare phenotype of CZS analyzed at exome-wide level to date. In addition, the present study was the first to include a detailed analysis of possible confounding covariates such as population stratification. Our results were all controlled for the time of ZIKV exposure during pregnancy, which has been clearly associated with CZS outcome.

In the present study, we provided a comprehensive screening for the role of children’s genetic background, where cases and controls had the same ZIKV exposure. Despite its limitations, this research highlighted promising candidates SNVs affecting pathways associated with CZS defects to be used for larger studies and functional validation. Our findings suggest that the complex phenotype of CZS may be mainly related to (i) the presence of known pathogenic variants in affected individuals, (ii) harmful variations associated with inborn genetic errors, and (iii) protective effect of SNVs in *DISP3* and *IL12RB2* genes. The potential protective associations for rs2076469 at the *DISP3* gene may play a role in CZS pathogenesis due to its association with neuronal proliferation and differentiation, both phenotypes are commonly altered in CZS. In addition, the presence of rare variants in *IL12RB2* gene sheds light on possible contributions of early immune-response to ZIKV infection. Description of genetic factors influencing the pathology of ZIKV infection have direct implications for development of therapeutic strategies as well as surveillance and even protection from ZIKV infection.

## Supporting information

S1 Fig Workflow diagram for the identification of pathogenic variants and association analyses for CZS phenotypes. The left panel describes the quality control process for the identification of damaging variants, and association analyses. The right panel showed the two association approaches performed (single variant and gene-based approaches) and the selection of genes for Enrichment Analysis.
(TIF)

S2 Fig Common and rare single nucleotide variants filtering strategies in WES data. A) Filtering strategy used to select common variants (MAF > 5%) in our cohort including non-coding, synonymous and non-synonymous SNVs. B) Rare variants prioritization, and C) Proportion of non-synonymous classification of rare SNVs in CZS patients.
(TIF)

S3 Fig Population Structure Analysis of Congenital Zika Syndrome (CZS) cohorts from Northeast and Southeast Brazil. A) Principal component Analysis of Zika cohorts from Paraiba and Rio de Janeiro inferred for 19,402 variants of the **CZS_cleandataset_LD_pruned** dataset. B) Bar plots show the ancestry proportion for three reference ancestries in each individual resulting from ADMIXTURE K = 3 on the **CZS_cleandataset_LD_pruned_1KGP**. Red, green and blue proportions are related to European, Native American and African ancestry proportions, respectively.
(TIF)

S4 Fig Q-Q plots for Firth’s regression analysis of the reduced dataset (n = 35). A) Q-Q plot for the unadjusted Firth’s regression analysis. B) Q-Q plot for the Firth’s regression analysis adjusted by timing of gestational exposure to ZIKV.
(TIF)

S5 Fig Manhattan and Q-Q plots for Firth’s regression analysis of the complete dataset (n = 40). (Top) Manhattan and Q-Q plot for the unadjusted Firth’s regression analysis. (Bottom) Manhattan and Q-Q plot for the Firth’s regression analysis adjusted by timing of gestational exposure to ZIKV.
(TIF)

S1 Table Patients description and sequencing information. (XLSX)

S2 Table Potentially pathogenic variants in CZS patients. (XLSX)

S3 Table Covariates list and its association with CZS phenotypes. (XLSX)

S4 Table Firth’s Logistic Modelling for CZS using the reduced dataset (n = 35). (XLSX)

S5 Table Firth’s Logistic Modelling for CZS using the complete dataset (n = 40). (XLSX)

S6 Table Gene-based association test using the reduced dataset (n = 35). (XLSX)

S7 Table Gene-based association test using the complete dataset (n = 40). (XLSX)

S8 Table Minor Allele frequencies for rare variants of *IL12RB2* gene in Worldwide populations. (XLSX)

S9 Table Gene Ontology overrepresentation analysis of the relevant genes obtained in this study. (XLSX)

S10 Table REACTOME analysis of the relevant genes obtained in this study. (XLSX)

S11 Table KEGG analysis of the relevant genes obtained in this study. (XLSX)

S1 Text Supplementary Material. (DOCX)
