# Supplementary figures and images for: Whole-exome sequencing reveals insights into genetic susceptibility to Congenital Zika Syndrome
Source: PLoS Negl Trop Dis. 2021 Jun 14;15(6):e0009507. doi: 10.1371/journal.pntd.0009507 (PMC8224898; doi:10.1371/journal.pntd.0009507)

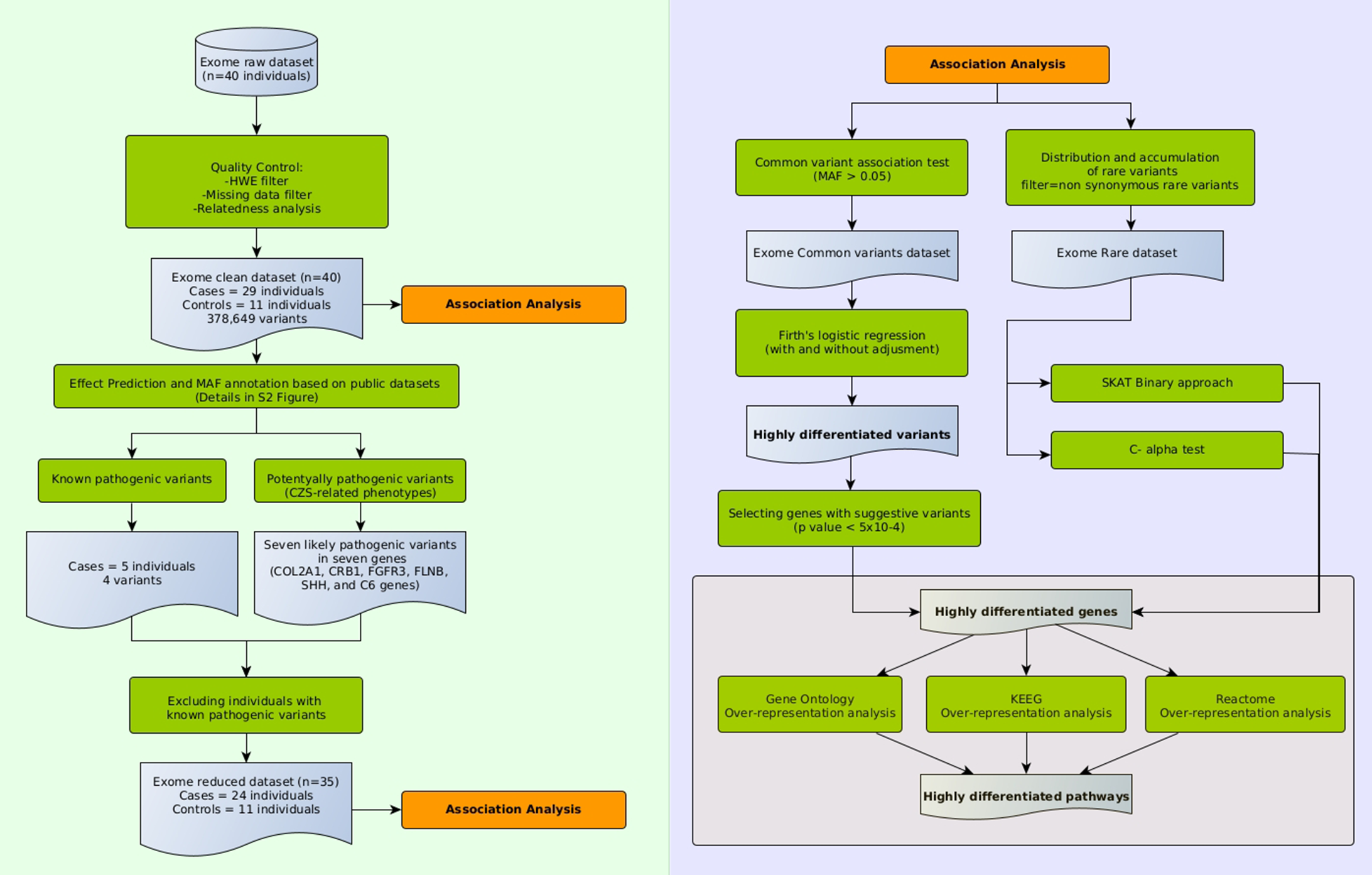

Supplement: S1 Fig — The left panel describes the quality control process for the identification of damaging variants, and association analyses. The right panel showed the two association approaches performed (single variant and gene-based approaches) and the selection of genes for Enrichment Analysis. (TIF) [file pntd.0009507.s001.tif]

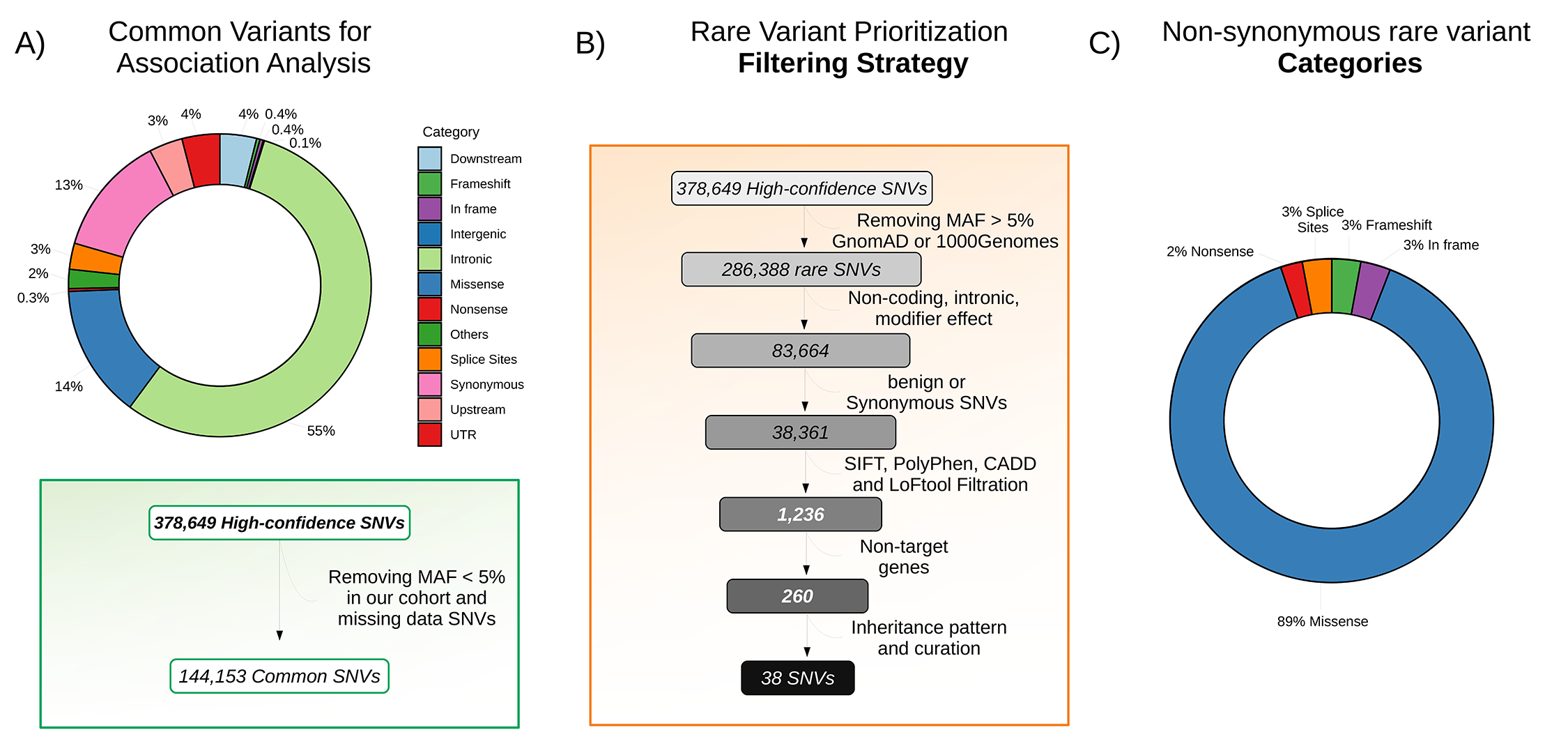

Supplement: S2 Fig — A) Filtering strategy used to select common variants (MAF > 5%) in our cohort including non-coding, synonymous and non-synonymous SNVs. B) Rare variants prioritization, and C) Proportion of non-synonymous classification of rare SNVs in CZS patients. (TIF) [file pntd.0009507.s002.tif]

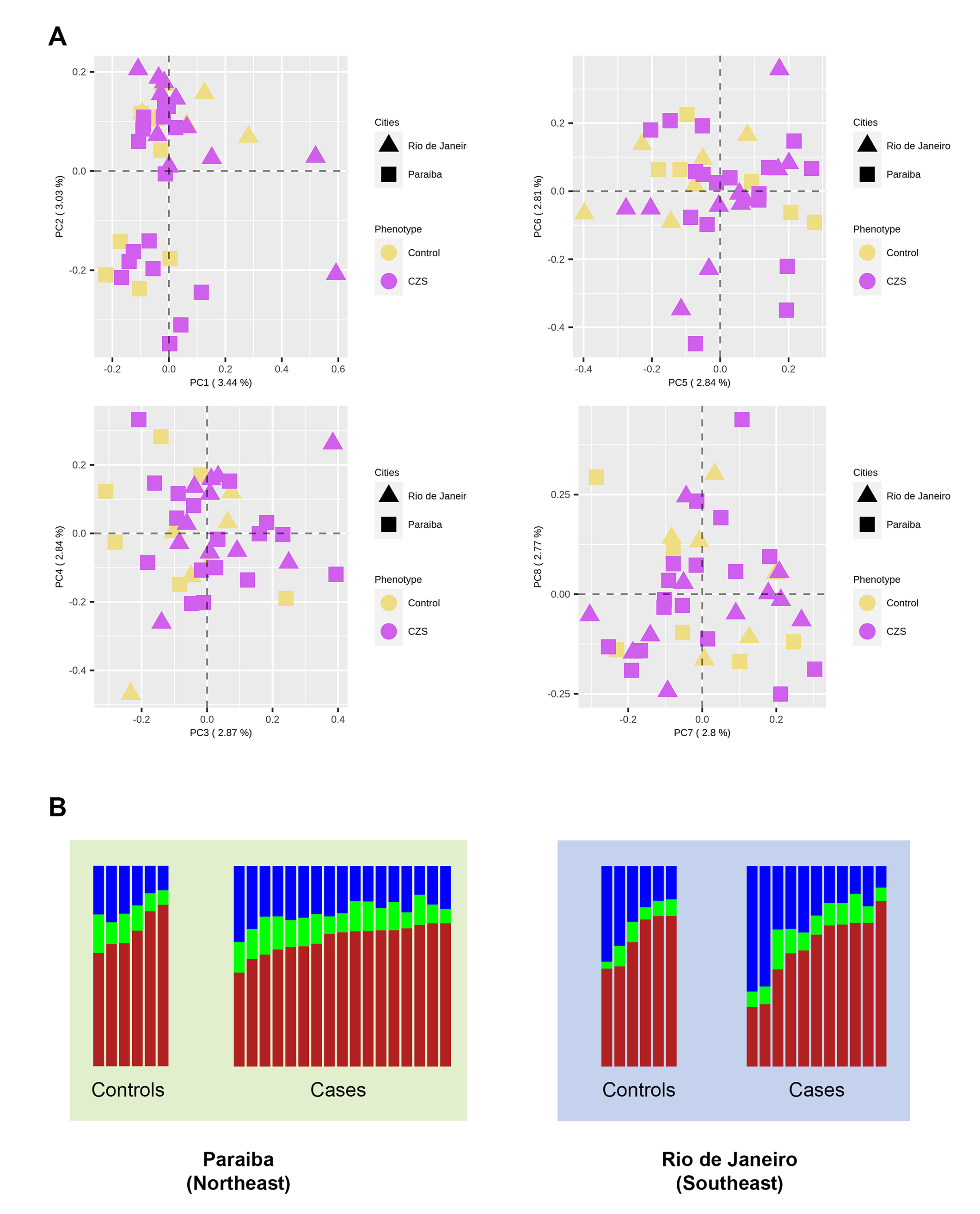

Supplement: S3 Fig — A) Principal component Analysis of Zika cohorts from Paraiba and Rio de Janeiro inferred for 19,402 variants of the CZS_cleandataset_LD_pruned dataset. B) Bar plots show the ancestry proportion for three reference ancestries in each individual resulting from ADMIXTURE K = 3 on the CZS_cleandataset_LD_pruned_1KGP. Red, green and blue proportions are related to European, Native American and African ancestry proportions, respectively. (TIF) [file pntd.0009507.s003.tif]

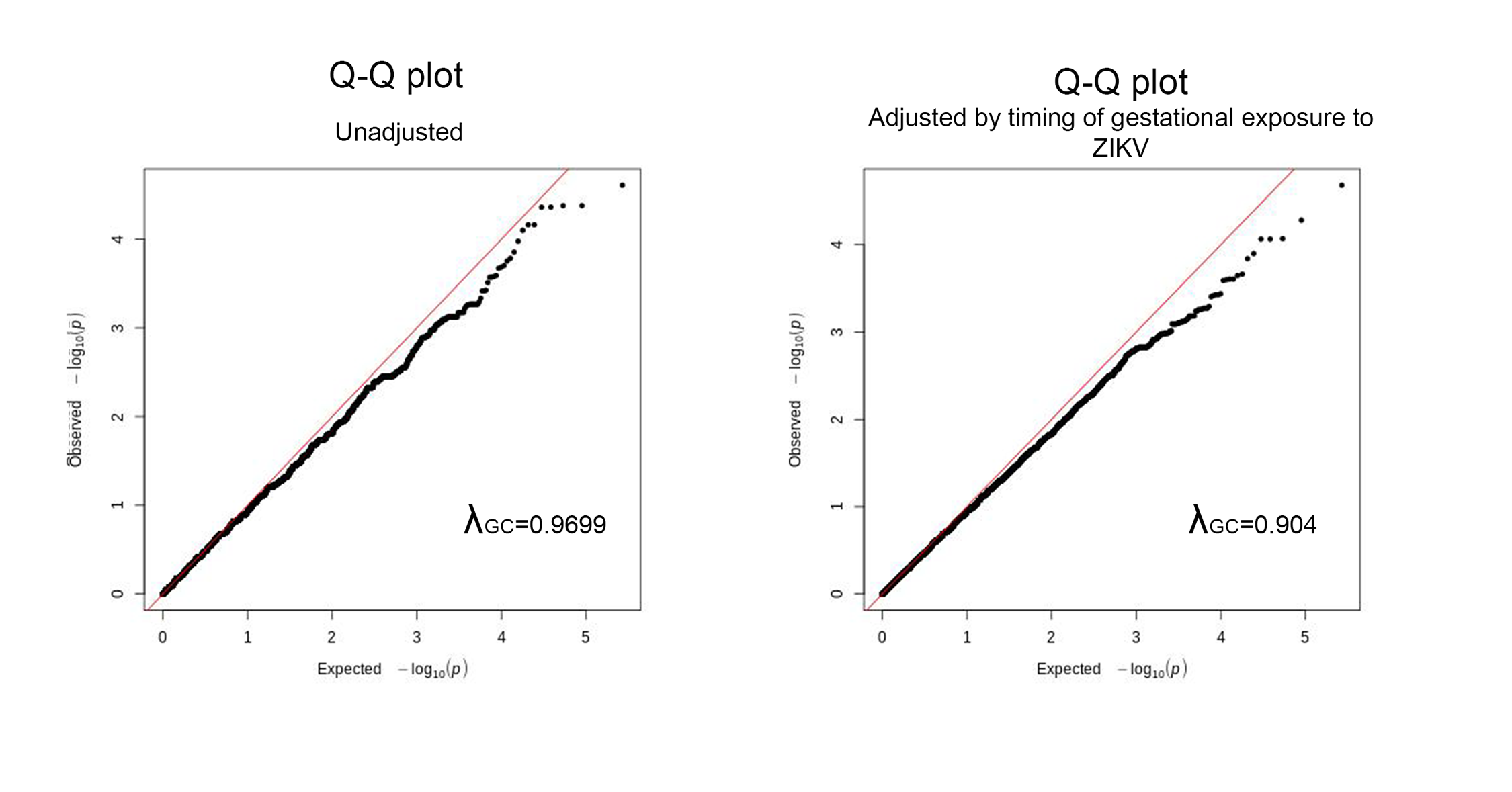

Supplement: S4 Fig — A) Q-Q plot for the unadjusted Firth’s regression analysis. B) Q-Q plot for the Firth’s regression analysis adjusted by timing of gestational exposure to ZIKV. (TIF) [file pntd.0009507.s004.tif]

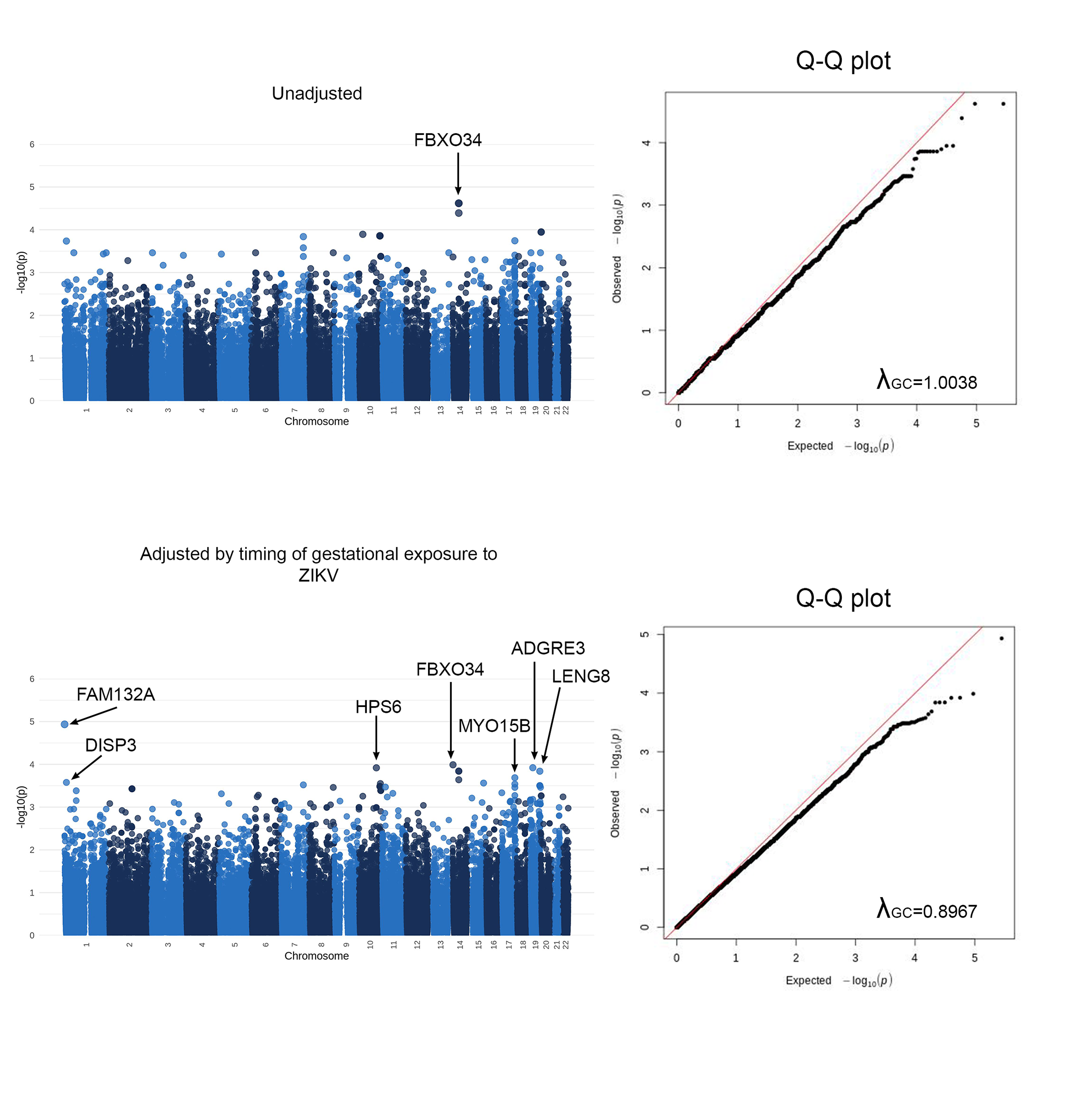

Supplement: S5 Fig — (Top) Manhattan and Q-Q plot for the unadjusted Firth’s regression analysis. (Bottom) Manhattan and Q-Q plot for the Firth’s regression analysis adjusted by timing of gestational exposure to ZIKV. (TIF) [file pntd.0009507.s005.tif]
