# Supplementary material for: Whole-exome sequencing reveals insights into genetic susceptibility to Congenital Zika Syndrome
Source: PLoS Negl Trop Dis. 2021 Jun 14;15(6):e0009507. doi: 10.1371/journal.pntd.0009507 (PMC8224898; doi:10.1371/journal.pntd.0009507)
Supplement: S1 Text — (DOCX) [file pntd.0009507.s017.docx]

Supplementary Material

Whole-exome sequencing reveals genetic insights into susceptibility to Congenital Zika Syndrome

**Running title: Genetics and Congenital Zika Syndrome**

Victor Borda^1¶^, Ronaldo da Silva Francisco Junior^1¶^, Joseane B Carvalho^1^, Guilherme L Morais^1^, Átila Duque Rossi^2^, Paula Pezzuto^2^, Girlene S. Azevedo^3^, Bruno L. Schamber-Reis^4^, Elyzabeth A. Portari^5^, Adriana Melo^3,4^, Maria Elisabeth L. Moreira^5^, Letícia C. Guida^5^, Daniela P. Cunha^5^, Leonardo Gomes^5^, Zilton F. M. Vasconcelos^5^, Fabio R. Faucz^6^, Amilcar Tanuri^2^, Constantine A. Stratakis^6^, Renato S. Aguiar^7&^*, Cynthia Chester Cardoso^2&^*, Ana Tereza Ribeiro de Vasconcelos^1&^*.

^1^Laboratório de Bioinformática, Laboratório Nacional de Computação Científica LNCC/MCTIC Petrópolis, Brazil

^2^Laboratório de Virologia Molecular, Instituto de Biologia, Universidade Federal do Rio de Janeiro, Rio de Janeiro, Brazil

^3^Instituto de Pesquisa Professor Amorim Neto, Campina Grande Brazil

^4^Faculdade de Ciências Médicas de Campina Grande, Núcleo de Genética Médica, Centro Universitário UniFacisa, Campina Grande, Brazil.

^5^Instituto Fernandes Figueira, Fiocruz, Rio de Janeiro, Brazil

^6^Section on Endocrinology and Genetics, *Eunice Kennedy Shriver* National Institute of Child Health and Human Development, National Institutes of Health, Bethesda, Maryland, USA

^7^Departamento de Genética, Ecologia e Evolução Instituto de Ciências Biológicas, Universidade Federal de Minas Gerais, Belo Horizonte, Brazil

^¶^ These authors contributed equally to this work.

^&^ These authors contributed equally to this work.

# *[atrv@lncc.br](mailto:atrv@lncc.br)(ATRV); [cynthiac@biologia.ufrj.br(CCC)](mailto:cynthiac@biologia.ufrj.br(CCC)); [santanarnt@gmail.com(RSA)](mailto:santanarnt@gmail.com(RSA));

# Supplementary Methods

### Kinship analysis

We performed an individual filtering based on kinship coefficient to infer cryptic relatedness that could mislead our association analysis. Since the Brazilian population has a trihybrid origin [[12]](https://paperpile.com/c/eGm8IF/XCi1c), we merged out our CZS_cleandataset_LD_pruned with 150 individuals from reference populations from 1000Genomes [[13]](https://paperpile.com/c/eGm8IF/vQdqC) for African, European and Native American ancestry. For African populations, we selected Yoruba in Ibadan, Nigeria (YRI) and Luhya in Webuye, Kenya (LWK). For Europeans, we selected Iberian population in Spain (IBS) and Utah Residents (CEPH) with Northern and Western European Ancestry (CEU) and For Native Americans, we selected individuals with high levels of Native American ancestry from the Peruvians from Lima (PEL) and Mexicans from Los Angeles, USA (MXL). Our final dataset for ancestry analysis consists of 19,402 autosomal independent variants for 190 individuals (CZS_cleandataset_LD_pruned_1KGP). We used this data set to calculate the kinship coefficient with REAP [[14]](https://paperpile.com/c/eGm8IF/wBh5B). We analyzed each pair of individuals and remove one of them if they share first or second degree [kinship coefficient (ϕ) > 0.125]. We did not find any kinship relationship in the dataset.

### Variant prioritizing

In order to identify novel variants with a potential pathogenic effect in our cohort, our framework of investigation interrogated variants with deleterious profiles according to computational predictors (SIFT, PolyPhen, CADD and LoFtool). We selected variants with zigozity profiles in each patient consistent with the phenotypic inheritance pattern of the gene (S1 Fig).

By querying the Human Phenotype Ontology (HPO; <https://hpo.jax.org/>) database, we analyzed genes related to abnormality of neuronal migration (HP:0002269), agyria (HP:0031882), arthralgia (HP:0002829) congenital contractures (HP:0002803), congenital microcephaly (HP:0011451), immunity (HP:0002715), lissencephaly (HP:0001339), ocular abnormalities (HP:0000496) and ventriculomegaly (HP:0002119) [[15]](https://paperpile.com/c/eGm8IF/fg5W0). We only include variants that met the following criteria SIFT = “deleterious”, PolyPhen = ”probably damaging”, CADD >= 20 and LoFtool <= 0.3.

### Population structure and covariables

We determine if population structure or a non-genetic variable explain the differentiation between cases and controls. For this reason, we performed association analyses among the phenotype (CZS/Control) and 10 first principal components, ancestry proportions, sex, and timing of gestational exposure (TGE) to ZIKV. For clinical variables, we include TGE to ZIKV, which was defined by the occurrence of exanthema. We considered this variable with three categorical outcomes: (0) non-information, (1) first trimester, and (2) after the first trimester.

For principal components and ancestry proportions, we perform a Principal component analysis (PCA) using SNPRelate [[16]](https://paperpile.com/c/eGm8IF/75pXO) and a genetic clustering using ADMIXTURE [[17]](https://paperpile.com/c/eGm8IF/z3Z6p), respectively. For both analyses, highly linked SNPs (r^2^ >0.1) and rare variants (MAF <= 0.05) were removed. We kept a dataset of 19,402 variants. For PCA, we analyze this dataset. For ADMIXTURE, we used the CZS_cleandataset_LD_pruned_1KGP that includes 1000Genomes References populations. Results were plotted on S2 Fig. The average ancestry proportions resulting from ADMIXTURE are described in S3 Table.

### Gene-based analysis using C-alpha and SKAT approaches

First, we only selected variants predicted as HIGH or MODERATE impacts according to the SnpEff classification, including nonsense, frameshift, splice sites, missense and in frame mutations. Next, we used variants with MAF < 5% in public databases (1000Genomes, Exac and GnomaD). The C-alpha tests the hypothesis that if the distribution of rare variants has no association with the phenotype then it follows a binomial distribution [[18]](https://paperpile.com/c/eGm8IF/VEGsK). The SKAT approach is a kernel association test that uses multiple regressions for the phenotype and the variants, and calculates p values by fitting a null model based on covariates [[19]](https://paperpile.com/c/eGm8IF/gHP5b). The C-alpha test was run using the AssotesteR package in R with 50,000 permutations and MAF = 0.05. For the SKAT approach, we applied SKAT Binary analysis to the effect of rare variants by under-weighting the effect of common variants [[20]](https://paperpile.com/c/eGm8IF/Rvi5e). We used the SKAT package on R [[20]](https://paperpile.com/c/eGm8IF/Rvi5e), we performed 1000 bootstrap replicates for the null model unadjusted and adjusted for covariates. We considered three covariates: Timing of gestational exposure to ZIKV and the first and second principal component. Also, we considered a maximum MAF of 5% for the function binary. All other parameters were set as default.

# References

1. [Langmead B, Salzberg SL. Fast gapped-read alignment with Bowtie 2. Nat Methods. 2012;9: 357–359.](http://paperpile.com/b/eGm8IF/rKmc9)

2. [Li H, Handsaker B, Wysoker A, Fennell T, Ruan J, Homer N, et al. The Sequence Alignment/Map format and SAMtools. Bioinformatics. 2009;25: 2078–2079.](http://paperpile.com/b/eGm8IF/V71Et)

3. [DePristo MA, Banks E, Poplin R, Garimella KV, Maguire JR, Hartl C, et al. A framework for variation discovery and genotyping using next-generation DNA sequencing data. Nat Genet. 2011;43: 491–498.](http://paperpile.com/b/eGm8IF/FUKEt)

4. [Van der Auwera GA, Carneiro MO, Hartl C, Poplin R, Del Angel G, Levy-Moonshine A, et al. From FastQ data to high confidence variant calls: the Genome Analysis Toolkit best practices pipeline. Curr Protoc Bioinformatics. 2013;43: 11.10.1–11.10.33.](http://paperpile.com/b/eGm8IF/lPXcv)

5. [Cingolani P, Platts A, Wang LL, Coon M, Nguyen T, Wang L, et al. A program for annotating and predicting the effects of single nucleotide polymorphisms, SnpEff: SNPs in the genome of Drosophila melanogaster strain w1118; iso-2; iso-3. Fly . 2012;6: 80–92.](http://paperpile.com/b/eGm8IF/aAnru)

6. [MacArthur J, Bowler E, Cerezo M, Gil L, Hall P, Hastings E, et al. The new NHGRI-EBI Catalog of published genome-wide association studies (GWAS Catalog). Nucleic Acids Res. 2017;45: D896–D901.](http://paperpile.com/b/eGm8IF/3OAAs)

7. [Landrum MJ, Lee JM, Benson M, Brown G, Chao C, Chitipiralla S, et al. ClinVar: public archive of interpretations of clinically relevant variants. Nucleic Acids Res. 2016;44: D862–8.](http://paperpile.com/b/eGm8IF/NCtMF)

8. [Sherry ST, Ward MH, Kholodov M, Baker J, Phan L, Smigielski EM, et al. dbSNP: the NCBI database of genetic variation. Nucleic Acids Res. 2001;29: 308–311.](http://paperpile.com/b/eGm8IF/ZQoKZ)

9. [1000 Genomes Project Consortium, Abecasis GR, Auton A, Brooks LD, DePristo MA, Durbin RM, et al. An integrated map of genetic variation from 1,092 human genomes. Nature. 2012;491: 56–65.](http://paperpile.com/b/eGm8IF/Rtw1J)

10. [Lek M, Karczewski KJ, Minikel EV, Samocha KE, Banks E, Fennell T, et al. Analysis of protein-coding genetic variation in 60,706 humans. Nature. 2016;536: 285–291.](http://paperpile.com/b/eGm8IF/qyqTd)

11. [Chang CC, Chow CC, Tellier LC, Vattikuti S, Purcell SM, Lee JJ. Second-generation PLINK: rising to the challenge of larger and richer datasets. Gigascience. 2015;4: 7.](http://paperpile.com/b/eGm8IF/QzB8T)

12. [Kehdy FSG, Gouveia MH, Machado M, Magalhães WCS, Horimoto AR, Horta BL, et al. Origin and dynamics of admixture in Brazilians and its effect on the pattern of deleterious mutations. Proc Natl Acad Sci U S A. 2015;112: 8696–8701.](http://paperpile.com/b/eGm8IF/XCi1c)

13. [1000 Genomes Project Consortium, Auton A, Brooks LD, Durbin RM, Garrison EP, Kang HM, et al. A global reference for human genetic variation. Nature. 2015;526: 68–74.](http://paperpile.com/b/eGm8IF/vQdqC)

14. [Thornton T, Tang H, Hoffmann TJ, Ochs-Balcom HM, Caan BJ, Risch N. Estimating kinship in admixed populations. Am J Hum Genet. 2012;91: 122–138.](http://paperpile.com/b/eGm8IF/wBh5B)

15. [Aguiar RS, Pohl F, Morais GL, Nogueira FCS, Carvalho JB, Guida L, et al. Molecular alterations in the extracellular matrix in the brains of newborns with congenital Zika syndrome. Sci Signal. 2020;13. doi:](http://paperpile.com/b/eGm8IF/fg5W0)[10.1126/scisignal.aay6736](http://dx.doi.org/10.1126/scisignal.aay6736)

16. [Zheng X, Levine D, Shen J, Gogarten SM, Laurie C, Weir BS. A high-performance computing toolset for relatedness and principal component analysis of SNP data. Bioinformatics. 2012;28: 3326–3328.](http://paperpile.com/b/eGm8IF/75pXO)

17. [Alexander DH, Novembre J, Lange K. Fast model-based estimation of ancestry in unrelated individuals. Genome Res. 2009;19: 1655–1664.](http://paperpile.com/b/eGm8IF/z3Z6p)

18. [Neale BM, Rivas MA, Voight BF, Altshuler D, Devlin B, Orho-Melander M, et al. Testing for an unusual distribution of rare variants. PLoS Genet. 2011;7: e1001322.](http://paperpile.com/b/eGm8IF/VEGsK)

19. [Wu MC, Lee S, Cai T, Li Y, Boehnke M, Lin X. Rare-variant association testing for sequencing data with the sequence kernel association test. Am J Hum Genet. 2011;89: 82–93.](http://paperpile.com/b/eGm8IF/gHP5b)

20. [Lee S, Fuchsberger C, Kim S, Scott L. An efficient resampling method for calibrating single and gene-based rare variant association analysis in case-control studies. Biostatistics. 2016;17: 1–15.](http://paperpile.com/b/eGm8IF/Rvi5e)

# 
